# Efficacy of *Lactobacillus fermentum* Isolated from the Vagina of a Healthy Woman against Carbapenem-Resistant *Klebsiella* Infections In Vivo

**DOI:** 10.4014/jmb.2103.03014

**Published:** 2021-08-25

**Authors:** Hanieh Tajdozian, Hoonhee Seo, Sukyung Kim, Md Abdur Rahim, Saebim Lee, Ho-Yeon Song

**Affiliations:** 1 Department of Microbiology and Immunology, College of Medicine, Soonchunhyang University, Chungnam, Cheonan 31151, Republic of Korea; 2 Probiotics Microbiome Convergence Center, Chungnam, Asan 31538, Republic of Korea

**Keywords:** Carbapenem-resistant *Enterobacteriaceae*, carbapenem-resistant *Klebsiella*, *Lactobacillus fermentum*, mouse model, mortality

## Abstract

Carbapenem-resistant *Enterobacteriaceae* (CRE) that produce *Klebsiella pneumoniae* carbapenemase are increasingly reported worldwide and have become more and more resistant to nearly all antibiotics during the past decade. The emergence of *K. pneumoniae* strains with decreased susceptibility to carbapenems, which are used as a last resort treatment option, is a significant threat to hospitalized patients worldwide as *K. pneumoniae* infection is responsible for a high mortality rate in the elderly and immunodeficient individuals. This study used *Lactobacillus fermentum* as a candidate probiotic for treating CRE-related infections and investigated its effectiveness. We treated mice with *L. fermentum* originating from the vaginal fluid of a healthy Korean woman and evaluated the *Lactobacilli*’s efficacy in preventive, treatment, non-establishment, and colonization mouse model experiments. Compared to the control, pre-treatment with *L. fermentum* significantly reduced body weight loss in the mouse models, and all mice survived until the end of the study. The oral administration of *L. fermentum* after carbapenemresistant *Klebsiella* (CRK) infection decreased mortality and illness severity during a 2-week observation period and showed that it affects other strains of CRK bacteria. Also, the number of *Klebsiella* bacteria was decreased to below 5.5 log_10_ CFU/ml following oral administration of *L. fermentum* in the colonization model. These findings demonstrate *L. fermentum*’s antibacterial activity and its potential to treat CRE infection in the future.

## Introduction

Probiotics are live microorganisms that can confer beneficial effects on the host when administered in adequate amounts [1-3]. It is important to understand that each probiotic strain independent of its genus and species has unique properties [4, 5]. The *Lactobacillus* and *Bifidobacterium* genera have recently become the focus of many research pieces as two of the most frequently applied probiotics [6]. Some studies have demonstrated that *Lactobacillus* has antibacterial effects against carbapenem-resistant *Enterobacteriaceae* (CRE), *Clostridium difficile*, *Escherichia coli*, *Shigella* spp., *Streptococcus mutans*, *Pseudomonas aeruginosa*, and *Staphylococcus aureus* [7-11]. The main mechanisms reported for the antimicrobial activity of probiotic strains are: adhering to the intestinal surfaces to inhibit the adhesion of pathogens, competing for nutrients and inhibiting the growth of pathogens, modifying intestinal immune responses, and reducing the risk of infection [12-15].

*Lactobacillus fermentum* is one of the dominant *Lactobacilli* in a healthy woman’s vaginal tract and intestine [16, 17]. Previous studies have defined *L. fermentum* as a possible probiotic candidate for protecting the intestine against pathogens and regulating microfloral balance [18, 19]. *L. fermentum* can inhibit intestinal pathogen growth by producing inhibitory compounds including H_2_O_2_, bacteriocin, and biosurfactants and improve intestinal bacteria flora by reducing *Clostridium perfringens* [20-22]. Moreover, other studies have reported that *L. fermentum* shows probiotic potential against gram-negative bacteria, including *E. coli*, *Salmonella* Typhimurium, and *Klebsiella pneumoniae* biofilm [23, 24].

CRE are gram-negative bacteria that seriously threaten public health, and infections due to these organisms are associated with significant morbidity and mortality [25]. Carbapenemase (including new Delhi metallo-β-lactamase (NDM), *K. pneumoniae* carbapenemase (KPC), and OXA-48) plays a major role in the antibiotic-resistant mechanism of CRE [26]. The increasing incidence of carbapenem-resistant *K. pneumoniae* (CRK) fundamentally alters the management of patients at risk of being colonized or infected by such microorganisms [27]. The crude mortality rate of diverse infections caused by CRK in patients ranges from 44% to 30.1% [28]. Moreover, invasive infections such as CRK bacteremia have shown striking crude and attributable mortality rates of 71.9% and 50%, respectively [28, 29]. At present, CRK is entirely resistant to all antibiotics, and there is no available drug to treat CRK infections [30]. Hence, there is an urgent need for a new drug candidate to produce antimicrobial drugs against CRK infections with improved efficacy.

Therefore, this study aimed to determine the in vivo antimicrobial activity of *L. fermentum* isolated from a healthy Korean woman’s vaginal fluid for preventing and treating CRK intestinal infections by inhibiting the pathogens’ growth and enhancing immunity in mouse models.

## Materials and Methods

### Carbapenem-Resistant *Klebsiella pneumoniae*

Clinical CRK isolates (SCHP191, SCHP192) were obtained from Soonchunhyang University Hospital Pathogenic Resource Bank and cultured in MacConkey broth (BD Difco, USA) at 37°C under aerobic conditions for 24 h. The cultures were streaked onto MacConkey agar (BD Difco) plates containing 10 mg/ml imipenem (Sigma-Aldrich, USA) and incubated at 30°C for 18 h. The isolated colonies were transferred to MacConkey broth and cultured at 30°C for 18 h. The bacterial culture (OD_600_ = 1.0, 2 × 10^9^ CFU/ml) was stocked in sterile glycerol and kept at -80°C. Antimicrobial susceptibilities were determined using the standard broth dilution method according to the CLSI guideline. Minimum inhibitory concentrations (MICs) were >1,025 mg/l for imipenem (Sigma-Aldrich), 1,025-512.5 mg/l for vancomycin (Sigma-Aldrich), 1,025-512.5 mg/l for kanamycin (Sigma-Aldrich), and >1,025 mg/l for metronidazole (Sigma-Aldrich). In the CRK mice infection experiment, CRK (SCHP191) was used in models 1,2, and 4, and CRK (SCHP191) and CRK (SCHP192) were used in model 3.

### Isolation of Probiotics from Vaginal Samples

A vaginal sample of a healthy woman was obtained from Soonchunhyang University Hospital and streaked onto MRS agar (BD Difco) plates followed by incubation at 30°C for 18 h. The isolated colonies were cultured in MRS broth (BD Difco) at 37°C under aerobic conditions for 24 h. The bacterial culture (OD_600_ = 1.0, 1 × 10^9^ CFU/ml) was stored at -80°C until use. Its 16S rRNA gene sequence was analyzed in-depth using a commercial sequencing service (BIOFACT Co., Korea). This study was accomplished in the Probiotics Microbiome Research Center in Soonchunhyang University, Korea. Ethics approval was obtained from the Ethics Committee of Soonchunhyang University Hospital (eIRB) (IRB No. 2019-10-017-005).

### 16S rRNA Gene Sequencing for Bacterial Identification

The 16S ribosomal RNA gene was amplified by PCR using two universal 16S rRNA gene primers (27F, 5′-AGA GTT TGA TCC TGG CTC AG-3′ / 1492R, 5′-GGT TAC CTT GTT ACG ACT T-3′) [31]. The PCR was carried out at 95°C initial denaturation for 3 min, followed by 30 cycles of 20 s at 95°C, 40 s at 56°C and 1 min 30 s at 72°C, and a final extension at 72°C for 5 min. The PCR product was purified using a PCR purification kit (BIOFACT Co., Korea) and sequenced with ABI PRISM 3730XL DNA Analyzer (Applied Biosystems, USA) using a BigDye Terminator v3.1 Cycle Sequencing Kit (Thermo Fisher Scientific, USA). Sequences were compared with the National Center for Biotechnology Information (NCBI) GenBank database using the Basic Local Alignment Search Tool (BLAST) search tool to find the closest matches.

### Ethics Committee and Experimental Animals

All animal experiments in this study were reviewed and approved by the Institutional Animal Ethics Committee of Soonchunhyang University (Korea), following the committee’s guidelines (SCH19-0053).

Nine-week-old specific pathogen-free (SPF) BALB/c female mice (Doo Yeol Biotech, Korea) were accommodated for one week before experiments. The mice were kept in individual cages with a 12 h/12 h light/dark cycle and relative humidity of 30 to 70% in an air-conditioned room (23 ± 2°C). They were provided free access to food and water *ad libitum*.

Four mice models were included in this study, and mice were randomly divided into three groups (*n* = 5 mice per group) for the experiments with models 1, 2, and 3. They were divided into four groups (*n* = 6 mice per group) for the experiment with model 4. The first and second mice models were used to evaluate the preventive potential and the curative effect, the third model for studying the effect of *L. fermentum* in a mouse model without the establishment of infection, and model 4 for determining the anti-infective activity of this strain in the colonization model. All animal experiments were performed per the World Health Organization recommendations in a biosafety level 2 facility (PMC animal room) at Soonchunhyang University.

### Study Design of in vivo *L. fermentum* Efficacy against CRK Multiple using Murine Infection Models

For the mouse infection models, the mice received a 200 μl solution of 0.2 M NaHCO_3_ (Sigma-Aldrich) orally on day 2 in models 1, 2, and 3 and on day 0 in model 4 to neutralize acidity and were challenged with CRK through the same route immediately after bicarbonate treatment. Cyclophosphamide (Sigma-Aldrich) at 450 mg/kg was administered intraperitoneally (200 μl/mouse) for experiments in models 1, 2, and 3, and a mixture of antibiotics (metronidazole, kanamycin, vancomycin each at a dose of 100 mg/kg) was orally administered once a day (200 μl/mouse) for model 4. Neutropenia was induced in models 1, 2, and 3 at 3 days before infection. The mice were induced with dysbiosis by administering the mixture of antibiotics on days -1 and -2 in model 4. In models 1, 2, and 3, survival rates and illness severity scores were measured to determine the effect of *L. fermentum* for reducing CRK infection. For endpoint analysis, fecal samples were collected from individual mice on days 2 and 5 for models 1 and 2 and days 2, 5, 8, and 14 continuously for the model 4 experiment. CFU per gram of stool was determined by plating each sample onto MacConkey agar containing 10 μg/ml of imipenem (~100 mg diluted in 1 ml of NaCl). All experiments were repeated at least two times, and each experiment was performed under the same conditions.

### Preparation of *Lactobacillus* Strain for Treatment

Briefly, the *L. fermentum* strain was cultivated in MRS broth at 37°C for 24 h. The stock vial was as described previously. After culture, it was washed with distilled water, resuspended in saline, and adjusted to an approximate concentration of 1.6 × 10^9^ CFU/mice/ml for experiments in models 1, 2, and 3 and 9 × 10^9^ CFU/ml in model 4. The number and viability of the *L. fermentum* strain in saline after 1 week were determined by culturing it on MRS plates under aerobic conditions, followed by enumerating the colonies after 48 h of incubation. Mice in treatment groups received saline containing *L. fermentum* through drinking water and oral gavage in models 1 and 2. They were administered only through drinking water in models 3 and 4. Mice in the control groups for all experiments received only sterile saline in a similar manner instead of the *lactobacillus* suspension under the same conditions as those in *lactobacillus*-treated groups.

### Acute Oral Toxicity Studies

For the acute toxicological studies, nine-week-old mice were randomly divided into two groups (*n* = 6 mice per group) and treated with saline drinking water (control) and drinking water containing *L. fermentum* at the concentration of 1.4 × 10^10^ CFU/ml. Animals were observed for clinical signs, mortality, and body weights for 14days following treatment. This study was performed according to the OECD Test Guidelines 423 with some modifications to test the acute oral toxicity of *L. fermentum* in the mouse models [32].

### CRK Mice Model 1 for Evaluating the Preventive Potential of *L. fermentum*

The preventative effect of *L. fermentum* on BALB/c mice was examined. Briefly, 1.6 × 10^9^ CFU/ml/mice of *L. fermentum* in 100 ml drinking water was given to the mice from 6 days before first CRK infection twice a week and was administered by oral gavage at a dose of 1.6 × 10^9^ CFU/mice/200 μl on day 3. Three days after neutropenia, infections were induced by oral administration of 200 μl CRK at 1 × 10^10^ CFU/mice on day 0 and 2 × 10^9^ CFU/mice on day 2. Infected mice received bicarbonate treatment before the second infection administration on day 2. To determine the number of viable CRK cells, stool samples were collected on days 2 and 5. MacConkey agar plates containing 10 μg/ml of imipenem were used to determine the number of viable CRK from serial dilutions of stool samples. The plates were incubated at 37°C aerobically for 24 h. The results are expressed as log_10_. The number of CFU was expressed as CFU/ml. The mice were monitored for 2 weeks consecutively after the first infection for survival rate, severity of illness score, and weight.

### CRK Mice Model 2 for Determining the Curative Effect of *L. fermentum* on CRK Lethal Sepsis

Mice were treated with 1.6 × 10^9^ CFU/ml/mice of *L. fermentum* in 100 ml drinking water from days 1 to 8 twice a week and at a dose of 1.6 × 10^9^ CFU/mice/200 μl on day 3 by oral gavage. Cyclophosphamide was injected on day -3, and infection was induced using 200 μl of the CRK suspension adjusted to 1 × 10^10^ CFU/mice on day 0 and 2 × 10^9^ CFU/mice on day 2. Infected mice received bicarbonate treatment before the second infection administration on day 2. Stool samples were collected on days 2 and 5, and 10 μl aliquots of serially diluted stool samples were inoculated onto MacConkey agar plates containing 10 μg/ml imipenem. These plates were incubated at 37°C overnight under aerobic conditions. The number of CFU/gram of stool was determined and expressed as log_10_ CFU/ml. Mice were observed for infection levels from day 0 until day 14 by measuring their body weights, recording their illness severity, and calculating their survival rate.

### CRK Mice Model 3 for Determining the Effect of *L. fermentum* in a Murine Model without the Establishment of Infection

In this examination, two different strains of CRK were used to evaluate the effect of *L. fermentum*. For each strain, mice were injected with cyclophosphamide on day -3 and infected with 200 μl of CRK suspension adjusted to 1 × 10^10^ CFU/mice on day 0 and 2 × 10^9^ CFU/mice on day 2. Infected mice received bicarbonate treatment before second infection administration on day 2. On day 0, from 2 h after oral infection until day 10, mice were treated with 1.6 × 10^9^ CFU/ml/mouse of *L. fermentum* in 100 ml drinking water. Subsequently, the mice’s body weights were measured, and infection levels on days 1–10 were monitored by recording illness severity and mortality.

### CRK Mice Model 4 for Determining the Anti-Infective Activity of *L. fermentum* in a Murine Model of Colonization

Mice received a single dose of a mixture of antibiotics (metronidazole, kanamycin, and vancomycin) on days -2 and -1 and were challenged by oral inoculation with 200 μl inoculum of CRK at 1 × 10^9^ CFU/mice on day 0 after receiving bicarbonate orally. The mice were treated with 9 × 10^9^ CFU/ml of *L. fermentum* in 150 ml drinking water twice a week for 2 weeks before CRK infection. To determine the CFU, serial dilution was performed, and a 10 μl aliquot of each dilution was inoculated onto MacConkey agar plates containing 10 mg/ml imipenem. The plates were incubated at 37°C overnight. Stool samples were collected on days 2, 5, 8, and 14 after oral infection, and the number of viable bacteria in the collected stool samples from mice was counted. Results are expressed as log_10_ CFU/ml.

### Determination of pH in Fecal Samples during *L. fermentum* Treatment

Nine-week-old BALB/c mice were divided into two groups (*n* = 6 mice in each group), one group received *L. fermentum* for 14 days, and the fecal pH was compared with the control group. The treatment group was given 100 ml saline containing 6 × 10^9^ CFU/ml of *L. fermentum* 3 times a week and was also administered twice a week using a zonde (6 × 10^9^ CFU/200 μl/mice). Control mice were exposed to saline like the treatment group. Fresh stool samples were collected before the start of *L. fermentum* treatment and on days 7, 11, and 14 after treatment. Each 100 mg of stool was vortexed with 1 ml of phosphate-buffered saline (PBS) and analyzed using an electronic pH meter (Mettler Toledo, Korea). This experiment was reviewed and approved by the Institutional Animal Ethics Committee of Soonchunhyang University (Asan, Korea) per the committee’s guidelines (SCH21-0019).

### Statistical Analysis

All data are presented as mean ± SEM (n¼5). They were log-transformed for each experiment. The statistical difference in the number of microorganisms from different treatment groups was determined. *p* < 0.05 was considered statistically significant.

## Results

### Identification and Acute Toxicity of the Isolated *Lactobacillus* Strain

**Fig. 1:**
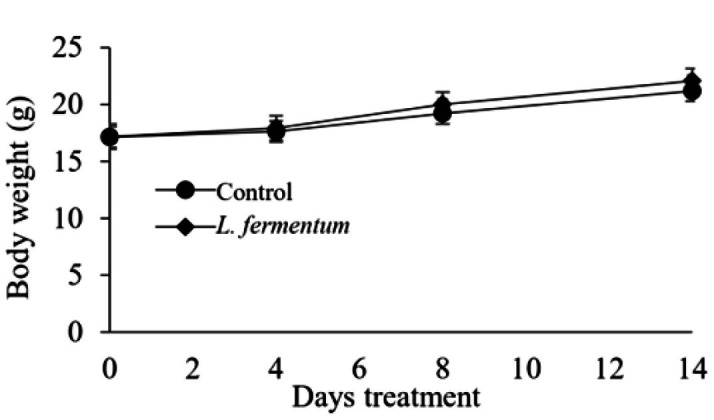
Results of acute toxicity assay of probiotics. Nine-week-old BALB/c mice were treated with *L. fermentum* twice a week. Weights of mice were recorded for 2 weeks.

The isolated vaginal *L. fermentum* was identified taxonomically by the robust 16S rRNA gene sequencing (Table 1). For the acute toxicity assay, nine-week-old mice were treated with *L. fermentum* at dose levels based on the maximum achievable concentration. We recorded their mortality, general appearance, and body weights until day 14. There was no mortality or abnormality observed during the acute oral toxicity test (Fig. 1).

### Pre-Treatment with *L. fermentum* before CRK Infection in Mice

**Fig. 2:**
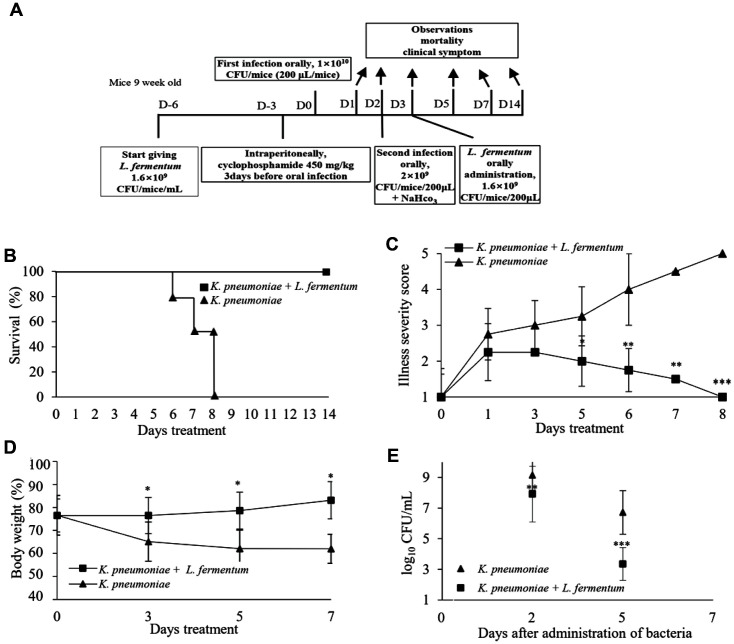
Prophylactic effect of probiotics in a CRK-infected mouse model. (**A**) *L. fermentum* was administered 6 days before infection to confirm the preventive effect of probiotics. (**B**) The survival rate of the mice was observed for 2 weeks postinfection. (**C**) Illness severity score was evaluated during 8 days. (1, healthy; 2, minimally ill; 3, moderately ill; 4, severely ill; 5, dead). (**D**) Body weight was measured for 7 days. (**E**) Stool samples were collected from individual infected mice, and a CFU test for CRK was performed. Statistical significance with controls was analyzed using unpaired Student's *t*-test (***p < 0.001; **p < 0.01; *p < 0.05).

Pre-treatment assay was performed to evaluate whether *L. fermentum* inhibited CRK infection (Fig. 2A). The pre-treatment results with *L. fermentum* showed that the mice treated with 1.6 × 10^9^ CFU/mice/ml of this strain before infection survived during the 14-day observation period (Fig. 2B). The sickness condition of mice was evaluated from day 0 until day 8. *L. fermentum*-treated mice showed a marked reduction in illness score compared to untreated mice (*p* < 0.001) (Fig. 2C). Additionally, untreated mice demonstrated significant body weight loss compared with that of the *L. fermentum*-treated mice (*p* < 0.05) (Fig. 2D). The preventative effect produced by *L. fermentum* on CRK infection in the mice was observed because *L. fermentum* significantly reduced CRK growth and decreased viable counts of CRK in stool samples to 3.2 log_10_ CFU/ml at 5 days post-infection (*p* < 0.001) (Fig. 2E). [Supplementary movie 1]

### Therapeutic Efficacy of *L. fermentum* in a Murine Model of CRK

**Fig. 3:**
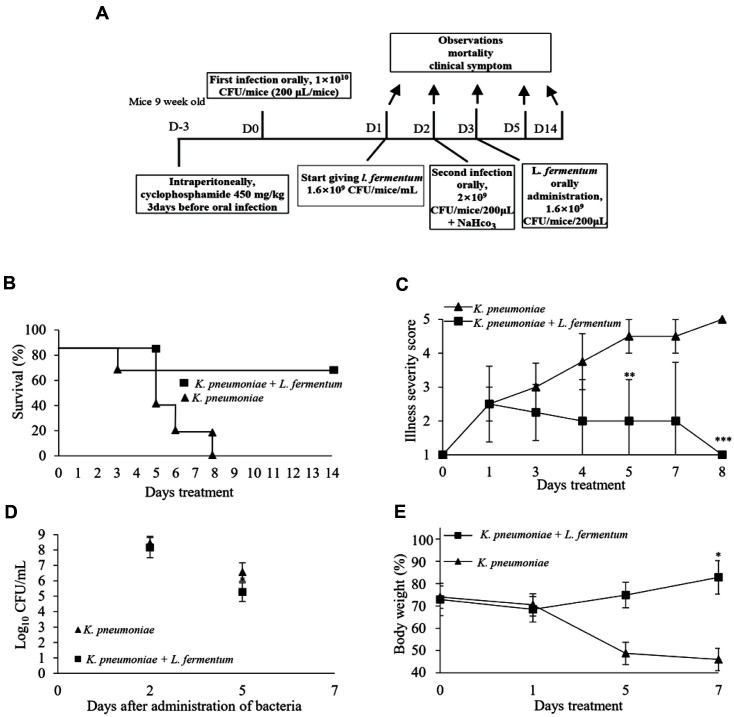
Therapeutic effect of probiotics on CRK in an established infectious mouse model. (**A**) In order to evaluate the therapeutic effect of probiotics on CRK infection, probiotics treatment was started one day after clinical signs appeared after infection. (**B**) Survival rates, (**C**) Illness severity score, and (**D**) weight changes were observed or analyzed during probiotic treatment after infection. (**E**) Stool samples were collected from individual infected mice, and a CFU test for CRK was performed. Statistical significance with controls was analyzed using unpaired Student's *t*-test (***p < 0.001; **p < 0.01; *p < 0.05).

The therapeutic effects of *L. fermentum* at a concentration of 1.6 × 10^9^ CFU/mice/ml on lethal CRK infections were evaluated in a mouse model (Fig. 3A). All mice infected with CRK in the untreated group died within 8 days after the first infection. In contrast, the survival rate was 75% for treated mice at 14 days post-infection (Fig. 3B). Untreated mice developed symptoms such as diarrhea and weakness after oral infection. Significant differences in illness scores between the treated and untreated groups were found from day 3 until day 8 (*p* < 0.001) (Fig. 3C). The untreated mice’s body weights became lower than those of mice in the *L. fermentum*-treated group during the study. Untreated mice showed severe weight loss until the endpoint at 7 days after the first CRK infection (*p* < 0.05)(Fig. 3D). CRK viable counts decreased to 5.2 log_10_ CFU/ml in the treated group stool samples, while the CRK growth rate remained high in the untreated group (*p* < 0.05) (Fig. 3E). [Supplementary movie 2]

### Antimicrobial Effect of *L. fermentum* in a Mouse Model without the Establishment of Infection

**Fig. 4:**
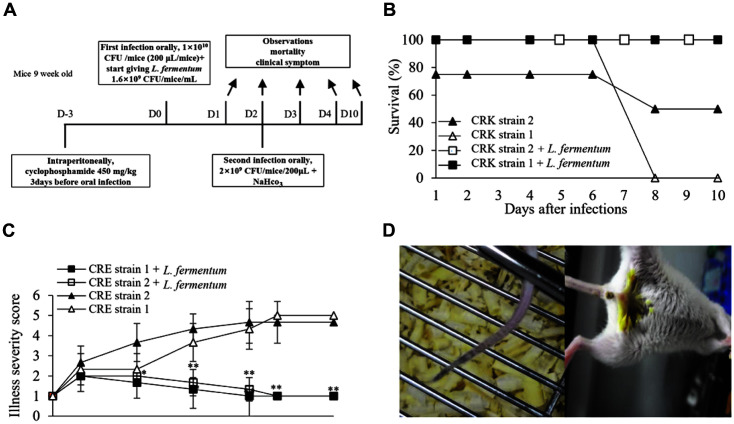
Effect of probiotics in a non-established CRK infection mouse model. The effect of probiotics was evaluated in a mouse model in which CRK infection was not established. (**B**) Survival rates and (**C**) disease severity scores following probiotic treatment were evaluated for infection of two clinically isolated CRK strains. (**D**) All mice treated with probiotics were alive and healthy, whereas mice not treated with probiotics developed blood clots (left) and diarrhea (right) and even died. Statistical significance with controls was analyzed using unpaired Student's *t*-test (**p < 0.01); *p < 0.05).

To evaluate the treatment effect produced in the mouse model without the establishment of infection, *L. fermentum* at a concentration of 1.6 × 10^9^ CFU/mice/ml was given through drinking water at 2 h after CRK administration on day 0 (Fig. 4A). Treated mice survived until the end of the study. Untreated mice that received CRK strain 2 showed 50% mortality by day 8, and all mice in the CRK strain 1 group died within 8 days (Fig. 4B). Mice in the untreated group showed higher scores of illness severity and disease symptoms, including weakness and diarrhea during the study, leading to significant differences between the treated and untreated groups (*p* < 0.01) (Fig. 4C). *L. fermentum* reduced both illness severity and mortality in the treated group of each CRK strain.

### Effects of *L. fermentum* on Intestinal Colonization in a Murine Model

Microbiological assessment of normal intestinal microflora in mice was performed by culturing uninfected mice fecal samples onto MacConkey agar plates containing 10 mg/ml imipenem during the study. No bacterial growth was observed on the agar plates after 24 h of incubation for all fecal samples. This result indicated an absence of CRE strains.

**Fig. 5:**
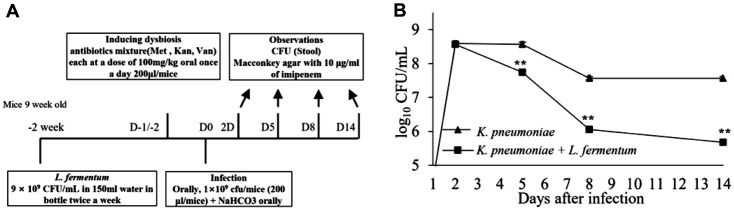
Decolonization effect of probiotics on intestinal CRK. (**A**) The decolonization effect of CRK colonized in the intestines of probiotics was evaluated in a CRK infection model that did not lead to death. CRK was infected after administration of a mixture of antibiotics (Met, methicillin; Kan, kanamycin; Van, vancomycin) to induce intestinal colonization of CRK. (**B**) Stool samples were continuously collected on days 2, 5, 8, and 14 during the 14-day observation period after infection, and CFU tests for CRK were performed. Statistical significance with controls was analyzed using unpaired Student's *t*-test (**p < 0.01, *p < 0.05).

The colonization experiment showed that CRK has the ability to colonize the intestines of mice. Mice received *L. fermentum* at a 9 × 10^9^ CFU/ml concentration before the CRK oral infection (Fig. 5A). The growth rate of CRK decreased in the group of mice treated with *L. fermentum* from after day 2. The mice were monitored for 2 weeks, and CFUs were measured during the study. *L. fermentum* reduced viable counts to 5.2 log_10_ CFU/ml in the treated group by day 14 (*p* < 0.01) (Fig. 5B). The numbers of CRK colonies were lower for stool samples of pre-treated mice than those in other groups until the end of the study. Therefore, there were significant differences in the CFU results between the pre-treated group (as a result of *L. fermentum* treatment) and the untreated group.

### Changes in pH of the Fecal Samples during *L. fermentum* Treatment

**Fig. 6:**
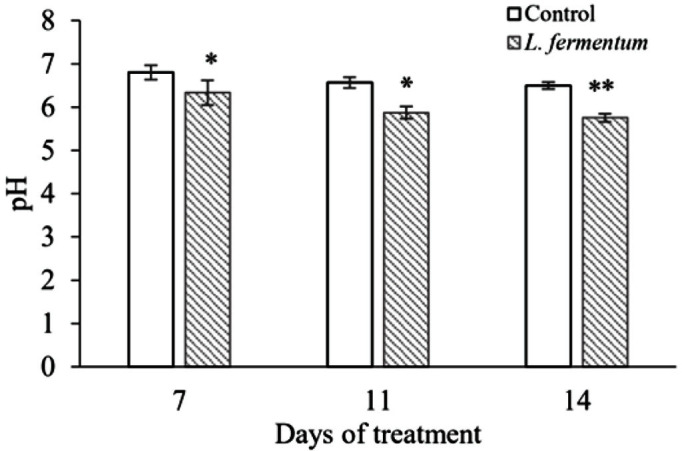
Changes in stool pH following oral administration of *L. fermentum* in mice. The pH value of the stool was measured while *L. fermentum* was administered orally for 2 weeks, and it was statistically compared with the control group through the unpaired Student's *t*-test (**p < 0.01, *p < 0.05).

To evaluate the effects of *L. fermentum* on fecal sample pH values, mice were treated with *L. fermentum* through oral gavage and drinking water. Before starting with *L. fermentum* treatment, the pH values were the same between the two groups and were found to be in the range of 6.9 ± 0.2. The pH values were determined in both the *L. fermentum*-treated and untreated groups on days 7, 11, and 14. The pH of the fecal sample significantly decreased in the *L. fermentum*-treated group and became lower than those of mice in the untreated group (*p* < 0.05) (Fig. 6A). The pH values were 6.80, 6.56, and 6.50 for the control group and 6.33, 5.87, and 5.75 in the *L. fermentum*-treatment group at days 7, 11, and 14, respectively.

## Discussion

Several experiments have shown that specific probiotic *Lactobacillus* spp. can inhibit intestinal infections by transit through the gastrointestinal tract, limiting the growth of pathogenic organisms and enhancing immunity [33-37]. Therefore, we expect probiotics to be used to solve antibiotic-resistant infections in the intestine by protecting it against pathogens. The main problem associated with antibiotic-resistant pathogens is the bacteria’s resistance to all antibiotics used [38]. Nowadays, CRE is one of the more urgent threats and *K. pneumoniae* is on the rise among CRE pathogens that can cause infections at different body sites, including the intestine [39-42]. Also, *K. pneumoniae*, a typical bacterium of the intestinal flora and feces, can potently induce intestinal inflammation by irregularly increasing its number in the colon. Intestinal carriage of CRK is associated with a high risk of infection [43-48].

In this study, we explored the antimicrobial potential of many probiotics, but only *L. fermentum* isolated from the vagina of a healthy Korean woman showed more significant activity against CRK infection. Vaginal *Lactobacillus* strains with known antagonistic properties against bacteria modulate the vaginal microbiota by various mechanisms: auto-aggregation, production of lactic acid, hydrogen peroxide, bacteriocins, biosurfactants, co-aggregation with pathogenic microorganisms, and adhesion to epithelial cells [49, 50]. Several investigators have reported that *L. fermentum* is an antimicrobial probiotic that can reduce the risk of infection and has the ability to reduce concentrations of pro-inflammatory factors, ameliorate colon cells, and reduce TNF-α levels in in vivo experiments [51, 52]. In vitro experiments have also shown that *L. fermentum* can co-aggregate with gastrointestinal pathogens and contribute to eliminating pathogens from the epithelia [53]. Other previous studies have also reported that whole cells of this *L. fermentum* and its acid supernatant exhibit an excellent ability to inhibit biofilm formation and *Klebsiella* growth. The acid supernatant of *L. fermentum* can also inhibit *Klebsiella* replication by producing high lactic acid and hydrogen peroxide levels. Thus, we evaluated this strain as a good candidate in this study.

In our view, *L. fermentum* might inhibit CRK, and the results of this study revealed the antimicrobial effect of *L. fermentum* against CRK. Several main findings were obtained by performing four different experiments using an infection model of mice in vivo.

Oral treatment with *L. fermentum* before infection markedly decreased illness severity and pathogenic infection induced in a mouse model and rescued mice from lethal CRK infections. Such observations indicate that *Lactobacillus* oral administration could stimulate the immune system to respond quickly to intestinal infections and support the potential preventive effects reported by recent studies of *Lactobacillus* against infections [54].

Throughout the evaluation of curative effects, a significant cure rate and a low peak illness severity score were observed for the *L. fermentum*-treated group, and the risk of mortality was also decreased for mice in the *L. fermentum*-treated group during the study period. Also, *L. fermentum* treatment showed a significant inhibitory effect against two different CRK strains in a non-establishment mouse model at only 8 days after CRK infection. Indeed, *Lactobacillus* treatment can possess a potent immunostimulatory effect to protect the host against *K. pneumoniae* infections and reduce such pathogen burdens based on previous reviews [55, 56].

According to previous studies, microbial gut overgrowth is hypothesized to be an important contributing factor to intestinal diseases [57]. To explore the *Lactobacillus*’ effect against CRK more comprehensively, we established a colonization mouse model and collected fecal samples from 2 days after oral administration. This study assessed the effectiveness of treatment with *L. fermentum* and showed this strain’s preventive ability against pathogenic intestinal colonization.

*L. fermentum* has been reported to increase organic acid and decrease pH in pig intestines [58]. In addition, administration of *Bifidobacterium breve* in mice increased organic acid concentration and decreased pH, resulting in a decrease in methicillin-resistant *S. aureus* (MRSA) [59]. In this study, oral administration of *L. fermentum* reduced the number of CRK in the mouse intestine and showed low fecal pH, suggesting a correlation between *L. fermentum* secretion of organic acids and their inhibitory effect on pathogens.

This study revealed that the *L. fermentum* strain possesses an excellent antimicrobial activity against CRK and characterized the strain’s probiotic properties. In conclusion, a vaginal *Lactobacillus* of a healthy woman was found to be a good candidate for treating CRK infections in the future. However, further studies are needed to identify more potential candidates and determine the biological mechanisms involved in the antimicrobial effect of *L. fermentum*, the optimal dosing, and possible therapeutic side effects.

## Supplemental Materials

Supplementary data for this paper are available on-line only at http://jmb.or.kr.

## Figures and Tables

**Table 1:** Identification of the isolated *L. fermentum* bacterial strain based on 16S rRNA gene sequence analysis and its close relatives published in DNA databases.

NCBI reference	Organism	Length	Score	Identities	Gaps
NR_113335.1	Lactobacillus fermentum strain NBRC 15885	1501	2752 bits (1490)	1495/1498 (99%)	0/1498(0%)
NR_104927.1	Lactobacillus fermentum strain CIP 102980	1502	2743 bits (1485)	1487/1488 (99%)	0/1488(0%)
NR_118978.1	Lactobacillus fermentum strain NCDO 1750	1381	2285 bits (1237)	1320/1382 (96%)	11/1382(1%)
NR_029084.1	Lactobacillus gastricus strain Kx156A7	1550	2410 bits (1305)	1436/1500 (96%)	6/1500(0%)
NR_028810.1	Lactobacillus ingluviei strain KR3	1506	2396 bits (1297)	1434/1500(96%)	9/1500 (1%)
NR_041566.1	Lactobacillus equigenerosi strain NRIC 0697	1519	2383 bits (1290)	1420/1483 (96%)	9/1483 (1%)
