# Supplementary material for: Efficacy of Lactobacillus fermentum Isolated from the Vagina of a Healthy Woman against Carbapenem-Resistant Klebsiella Infections In Vivo
Source: J Microbiol Biotechnol. 2021 Aug 25;31(10):1383–92. doi: 10.4014/jmb.2103.03014 (PMC9705860; doi:10.4014/jmb.2103.03014)
Supplement: Supplementary file 1 [file jmb-31-10-1383-supple.pdf]

J. Microbiol. Biotechnol.
https://doi.org/10.4014/jmb.2103.03014

A B

Supplementary movie 1
Preventive model

Supplementary movie 2
Therapeuticmodel

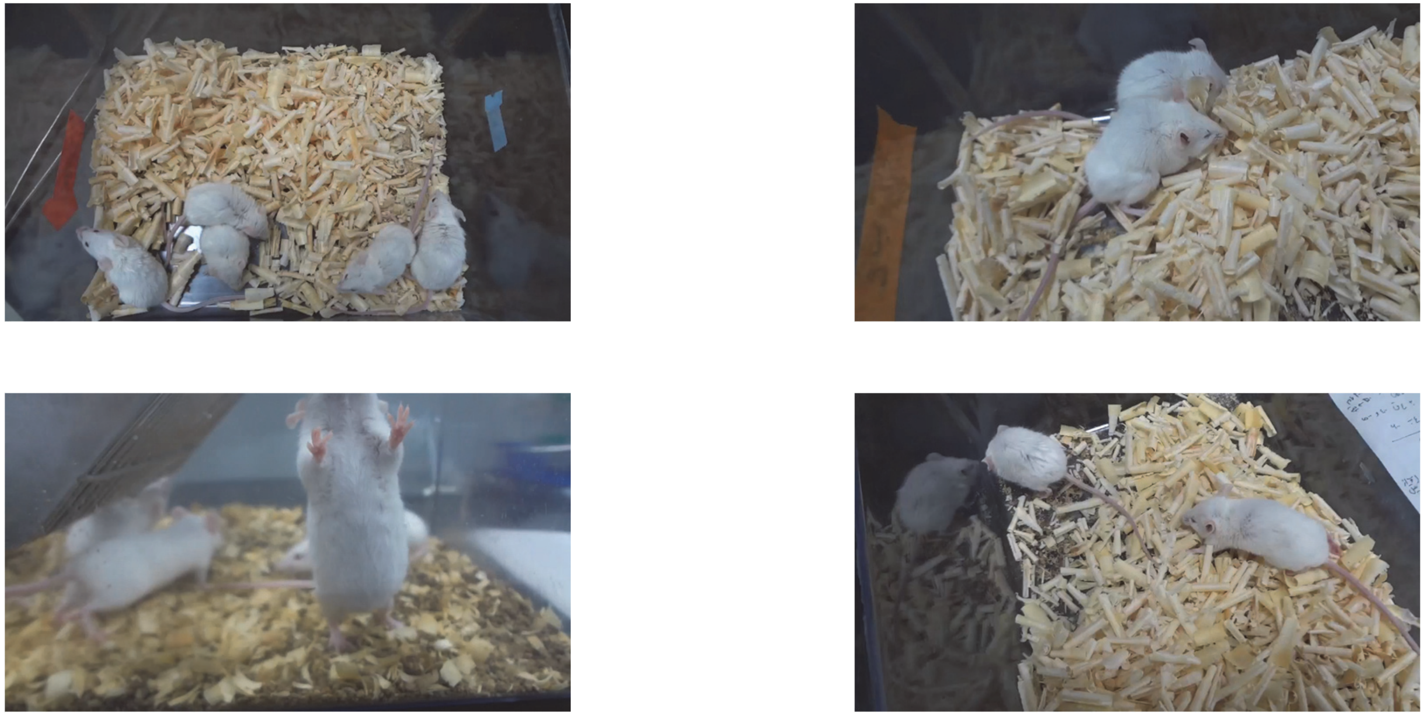

Supplementary movie 1 and 2.

1. Preventive model (A) *L. fermentum* treated mice (B) untreated mice
2. Therapeutic model model (A) *L. fermentum* treated mice (B) untreated mice
